# Exogenous Melatonin Increases Root Yield and Its Medicinal Quality of *Glycyrrhiza glabra* Under Drought Stress

**DOI:** 10.3390/plants15010075

**Published:** 2025-12-26

**Authors:** Hui Tian, Minghao Zhou, Miao Ma

**Affiliations:** Xinjiang Production and Construction Corps Key Laboratory of Oasis Town and Mountain-Basin System Ecology, Key Laboratory of Xinjiang Phytomedicine Resource Utilization, Ministry of Education, College of Life Sciences, Shihezi University, Shihezi 832003, China; tianhui@stu.shzu.edu.cn (H.T.); 18900720799@163.com (M.Z.)

**Keywords:** licorice, plant growth regulator, abiotic stress, root biomass, glabridin, glycyrrhizic acid

## Abstract

*Glycyrrhiza glabra* L. is an economically significant plant that naturally grows in arid regions and is widely used in the cosmetics, food, and pharmaceutical industries. Its roots are the economically important part. However, it has weak drought tolerance during the seedling stage, and water scarcity has become a major limiting factor for improving the yield and quality of cultivated licorice. Therefore, this study conducted a pot experiment in which melatonin was applied via root irrigation to examine its effects on alleviating drought stress in *G. glabra* seedlings and on enhancing the yield and quality of its valuable parts. The results showed that under drought conditions, applying 100 μM melatonin yielded the most significant improvements in both yield and quality. Specifically, melatonin treatment increased root biomass by 138.10% and significantly boosted the levels of key bioactive compounds, including glycyrrhizic acid, liquiritin, glabridin, liquiritigenin, and isoliquiritigenin by 60.51%, 72.08%, 182.03%, 83.86%,and 30.68%, respectively. This study uniquely combined the Mantel test and random forest modeling for a comprehensive analysis of the experimental data. The analysis indicated that these effects were attributable to exogenous melatonin, which markedly enhanced antioxidant enzyme activities in *G. glabra* seedlings and reduced membrane lipid peroxidation products, thereby strengthening their antioxidant defense capabilities. Additionally, melatonin promoted the accumulation of osmotic adjustment substances and effectively improved photosynthetic performance. Our research provides a scientific basis for increasing both the quality and yield of *G. glabra* under drought conditions.

## 1. Introduction

According to the Global Dryland Ecosystem Programme (Global-DEP), arid regions currently occupy approximately 41% of the Earth’s terrestrial surface and continue to expand [1]. It is projected that by the year 2100, nearly half of the global land area may be transformed into dryland ecosystems [2]. Water scarcity has emerged as a critical abiotic factor constraining the sustainable development of agriculture in arid regions, imposing substantial challenges to plant physiological and metabolic processes [3]. Drought stress disrupts plant physiological processes primarily by inducing excessive accumulation of reactive oxygen species (ROS), which initiates lipid peroxidation and damages cell membrane integrity [4]. This cascade not only disrupts membrane integrity but also accelerates the degradation of photosynthetic pigments and induces stomatal closure, thereby markedly suppressing photosynthetic efficiency. As a result, plant growth is severely impaired, and crop yield is substantially reduced [5]. In addition, drought stress profoundly alters carbon allocation within plants, redirecting greater carbon resources toward maintaining basic metabolism and stress responses, such as the synthesis of osmoprotectants and antioxidant enzymes, at the expense of secondary metabolism [6]. This shift diminishes the availability of precursors for biosynthesis required of secondary metabolites, including phenolics, flavonoids, and terpenoids, thereby impairing their accumulation. Although plants possess intrinsic drought-responsive mechanisms, these are often inadequate under prolonged or extreme drought condition to counteract the deleterious effects [7]. Therefore, identifying effective exogenous interventions to mitigate drought stress is of critical importance for improving crop yield and quality in the arid region.

*Glycyrrhiza glabra* L. is a perennial herbaceous leguminous plant characterized by a well-developed taproot system, pinnately compound leaves, and purple to bluish racemose flowers that develop into oblong pods. The species is widely distributed across arid regions in many countries of Europe and Asia [8,9]. Its dried root has long been utilized as a traditional medicinal material, being rich in pharmacologically active compounds such as glycyrrhizic acid and glycyrrhetinic acid, which exert diverse multiple therapeutic effects, including heat-clearing, detoxification, hepatoprotection and cough relief [10,11]. Moreover, these compounds function as highly efficient natural sweeteners, exhibiting sweetness levels approximately 100 and 300 times greater than that of sucrose, respectively [12]. Consequently, *G. glabra* is extensively utilized in both the pharmaceutical and food industries. In addition, it is a important raw material of raw material for modern cosmetics; among its active constituents, glabridin is particularly notable for its potent whitening and antioxidant properties, earning it the reputation of the “Skin Whitening Gold” [13]. Due to excessive exploitation and habitat loss, *G. glabra* is facing a critical crisis, its numerous wild populations have apparently become extinct, and the sizes of many surviving populations have been markedly reduced, rendering wild resources insufficient to meet the growing human demand [14]. Therefore, the practical development of cultivation techniques for *G. glabra* has become increasingly urgent. Although mature *G. glabra* plants are commonly distributed in arid regions and exhibit strong drought tolerance, their seedlings are considerably more susceptible to drought stress. This vulnerability often results in poor survival, slow growth, low yield and reduced secondary metabolite content under cultivation conditions [15,16]. Consequently, the high sensitivity of its seedlings to drought stress represents a major bottleneck limiting both high-yield cultivation and quality improvement.

In recent years, the application of exogenous substances has emerged as an important strategy for enhancing crop tolerance to drought stress and improving yield and quality [17,18]. Melatonin (MT), a naturally occurring indole compound, is widely distributed in plants [19]. It plays a pivotal role in plant responses to abiotic stress conditions [20]. Melatonin effectively mitigate cellular damage caused by oxidative stress by scavenging reactive oxygen species (ROS), activating antioxidant enzyme systems such as superoxide dismutase (SOD), catalase (CAT), and ascorbate peroxidase (APX), and regulating the levels of non-enzymatic antioxidants, including glutathione (GSH) and ascorbic acid (AA) [21,22]. These mechanisms collectively enhance plant tolerance to abiotic stresses such as drought and salinity–alkalinity. In addition, melatonin has been shown to promote root development and the accumulation of secondary metabolites, thereby contributing to improved drought resistance and yield enhancement in leguminous plants [23]. However, its regulatory effects are concentration-dependent, and excessive application may exert adverse impacts on plant physiology. Previous studies have indicated that high concentrations of melatonin can disrupt endogenous hormone homeostasis, inhibit root elongation, trigger premature senescence responses, and exacerbate oxidative stress, ultimately resulting in membrane damage and metabolic imbalance [24]. Therefore, elucidating the mechanisms by which melatonin functions under stress conditions and determining its optimal application dosage are critically important for fully harnessing its physiological regulatory potential to improve plant stress tolerance.

It has been reported that mild drought treatment can enhance the concentrations of certain active compounds in the roots of *G. glabra*; however, it also markedly reduces the root biomass, resulting in a substantial decline in the total amount of active constituents per plant [25]. This trade-off negatively affects both the economic value and the sustainable utilization of this medicinal herb. Accordingly, we aimed to identify an approach that can enhance the root yield of the licorice under water-deficient conditions, while maintaining or increasing the levels of its active constituents. In this study, we systematically evaluated the effects of exogenous melatonin application on the growth, physiological and biochemical traits, and root secondary metabolite accumulation of *G. glabra* seedlings under drought stress. We further identified the optimal application dosage of the melatonin, and employed Mantel test and random forest modeling to uncover the key factors influenced the root yield and quality under drought conditions.

## 2. Results

### 2.1. Effects of Exogenous Melatonin on the Growth of G. glabra Seedlings

**Figure 1:**
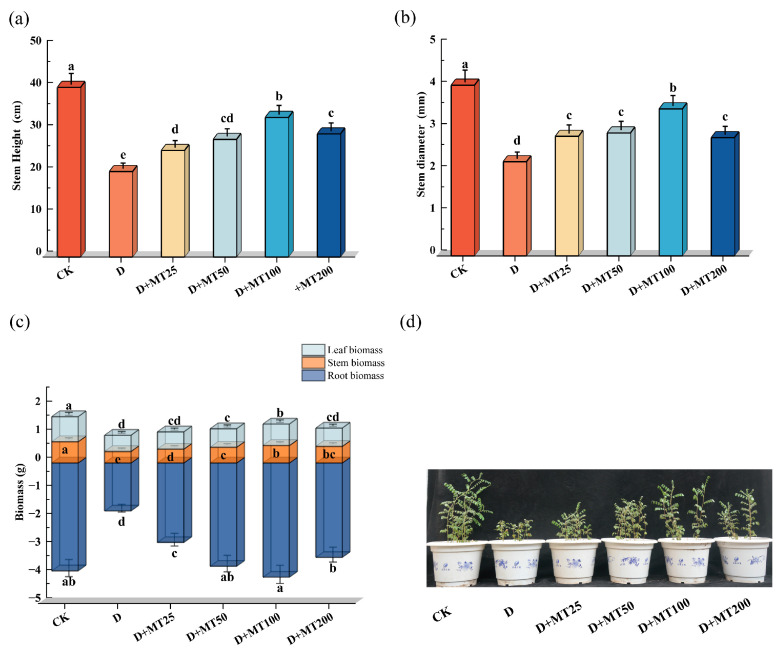
Effects of exogenous melatonin (MT) on the growth of *G. glabra* seedlings under drought treatment. (**a**) Stem height; (**b**) Stem diameter; (**c**) Root, stem, and leaf biomass; (**d**) Growth phenotype. Different lowercase letters indicate significant differences between different treatments, (Mean ± SD, *p* < 0.05).

Drought stress (D) significantly inhibited the growth of *G. glabra* seedlings. Relative to the control group (CK), seedlings under drought stress exhibited restricted development, characterized by reduced plant height, thinner stems, leaf yellowing, wilting, and partial abscission. Among all the treatments, seedlings in the D + MT100 group, treated with 100 μM melatonin, showed the most pronounced recovery, with greener and fuller leaves, sturdier stems, and enhanced vigor, clearly indicating the alleviation of drought-induced damage (Figure 1a,d). Under drought condition, stem height, stem diameter, and biomass of roots, stems, and leaves of the seedlings decreased by 49.63%, 44.84%, 55.52%, 45.79% and 35.39%, respectively, compared with the control group (CK). However, irrigation with melatonin significantly improved seedling growth under drought stress. At a concentration of 100 μM melatonin, the stem height, stem diameter, and biomass of roots, stems, and leaves increased by 63.37%, 56.15%, 138.10%, 51.83% and 32.56%, respectively, compared with the drought treatment group (D), and the root biomass of the group was slight higher than that of the CK (Figure 1a–c).

**Figure 2:**
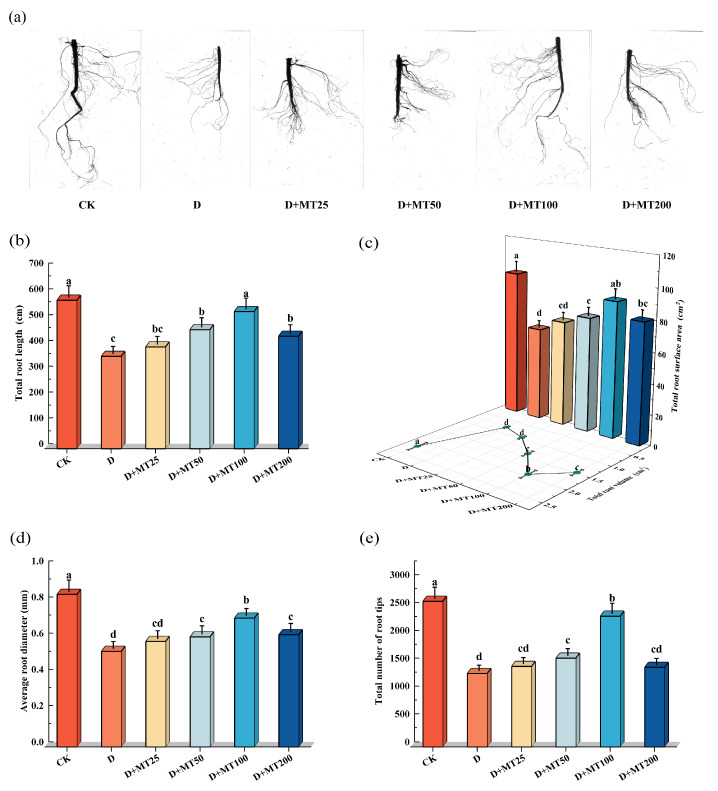
Effects of exogenous melatonin (MT) on the root morphological parameters of *G. glabra* seedlings under drought stress. (**a**) Root morphology; (**b**) Total root length; (**c**) Total root surface area and total root volume; (**d**) Average root diameter; (**e**) Total number of root tips. Different lowercase letters indicate significant differences between different treatments, (Mean ± SD, *p* < 0.05).

Drought treatment significantly inhibited root system development in *G. glabra*. Compared with CK, seedlings under the D treatment exhibited thinner primary roots, markedly reduced lateral root numbers, sparse branching, and a notably restricted root spread, indicating substantial suppression of root growth under stress conditions. In contrast, the application of exogenous melatonin greatly improved root system development. In particular, treatment with 100 μM melatonin resulted in the most pronounced effects, significantly increasing primary root length and enhancing both the density and length of lateral roots (Figure 2a).

Drought treatment significantly reduced the total root length, total root surface area, total root volume, average root diameter, and total number of root tips of *G. glabra* seedlings (Figure 2b–e). Compared with CK, these parameters decreased by 37.82%, 36.26%, 64.83%, 37.40% and 49.66%, respectively. Application of exogenous melatonin effectively alleviated the inhibitory effects of drought stress on root system development. In particular, irrigation with 100 μM melatonin markedly promoted root morphological traits, resulting in increases of 48.56%, 44.81%, 150.86%, 46.49% and 78.33% in total root length, surface area, volume average root diameter, and total number of root tips, respectively, compared with Drought treatment. And the total length and surface area of roots of the seedlings under the D + MT100 treatment were nearly equal to those of the CK (*p* > 0.05).

### 2.2. Effects of Exogenous Melatonin on Photosynthetic Gas Exchange Parameters and Leaf Chlorophyll Content

**Figure 3:**
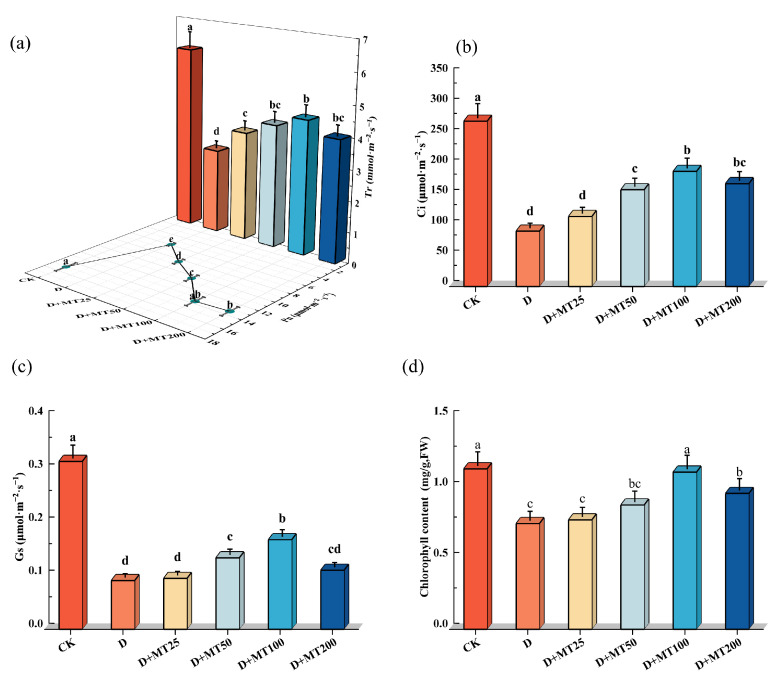
Effects of exogenous melatonin (MT) on photosynthetic gas exchange parameters and leaf chlorophyll content under drought stress. (**a**) Net photosynthetic rate (Pn) and transpiration rate (Tr); (**b**) Intercellular CO_2_ concentration (Ci); (**c**) Stomatal conductance (Gs); (**d**) Chlorophyll content. Different lowercase letters indicate significant differences between different treatments, (Mean ± SD, *p* < 0.05).

Drought treatment significantly reduced photosynthetic gas exchange parameters and chlorophyll content of the leaves. Compared with CK, seedlings under the drought treatment exhibited marked decreases in Pn, Gs, Ci, and Tr by 60.83%, 70.97%, 66.45% and 53.98%, respectively. However, application of 100 μM melatonin effectively alleviated these reductions, resulting in increases of 130.78%, 83.81%, 107.78% and 58.29% for the above four parameters compared with the D treatment. Drought stress resulted in a 34.09% reduction in chlorophyll content in the leaves compared with CK. However, D + MT100 treatment increased chlorophyll content by 48.61% compared with the drought-stressed group (D), as high as the level observed in the CK group (Figure 3).

### 2.3. Effects of Exogenous Melatonin on Leaf Relative Water Content

**Figure 4:**
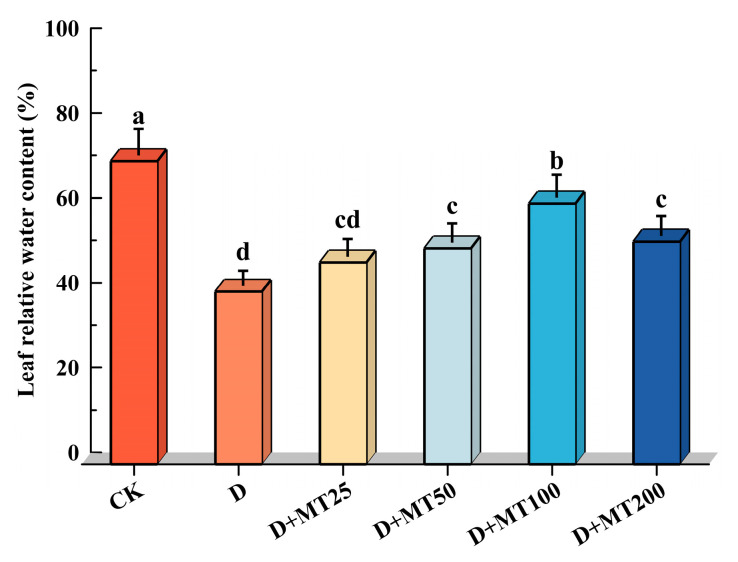
Effects of exogenous melatonin (MT) on the RWC in the leaves of *G. glabra* seedlings under drought stress. Different lowercase letters indicate significant differences between different treatments, (Mean ± SD, *p* < 0.05).

Relative water content (RWC) is a key physiological indicator reflecting the water status and drought tolerance of plants. In this study, drought treatment (D) significantly reduced the RWC of *G. glabra* by 42.99% compared with CK. Among the treatments with different concentrations of melatonin, the treatment of irrigation with 100 μM melatonin produced the most pronounced positive effect, increasing RWC by 51.09% compared with the D treatment (Figure 4).

### 2.4. Effects of Exogenous Melatonin on the Antioxidant Enzyme System

**Figure 5:**
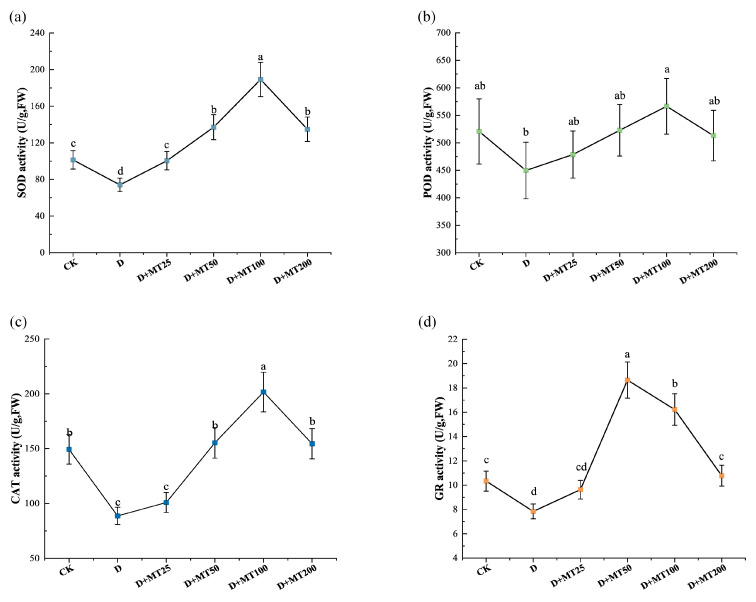
Effects of exogenous melatonin (MT) on the antioxidant enzyme activity in the leaves of *G. glabra* seedlings under drought stress. (**a**) Superoxide dismutase (SOD) activity; (**b**) Peroxidase (POD) activity; (**c**) Catalase (CAT) activity; (**d**) Glutathione reductase (GR) activity. Different lowercase letters indicate significant differences between different treatments, (Mean ± SD, *p* < 0.05).

To investigate the effects of exogenous melatonin application on antioxidant enzyme activities in *G. glabra* under drought stress, the activities of superoxide dismutase (SOD), peroxidase (POD), catalase (CAT), and glutathione reductase (GR) were measured. Compared with CK, drought treatment significantly reduced the activities of the above 4 enzyme by 26.93%, 13.65%, 40.61% and 24.12%, respectively (Figure 5). Melatonin treatments markedly enhanced their activities, at a melatonin concentration of 100 μM (D + MT100 treatment), the activities of SOD, POD, and CAT reached their maximum levels, showing increases of 155.43%, 25.96% and 127.29% (Figure 5a–c), respectively, compared with the D treatment. GR activity peaked at the treatment of D + MT50, followed by D + MT100 (Figure 5d).

### 2.5. Effects of Exogenous Melatonin on Membrane Permeability and Lipid Peroxidation

**Figure 6:**
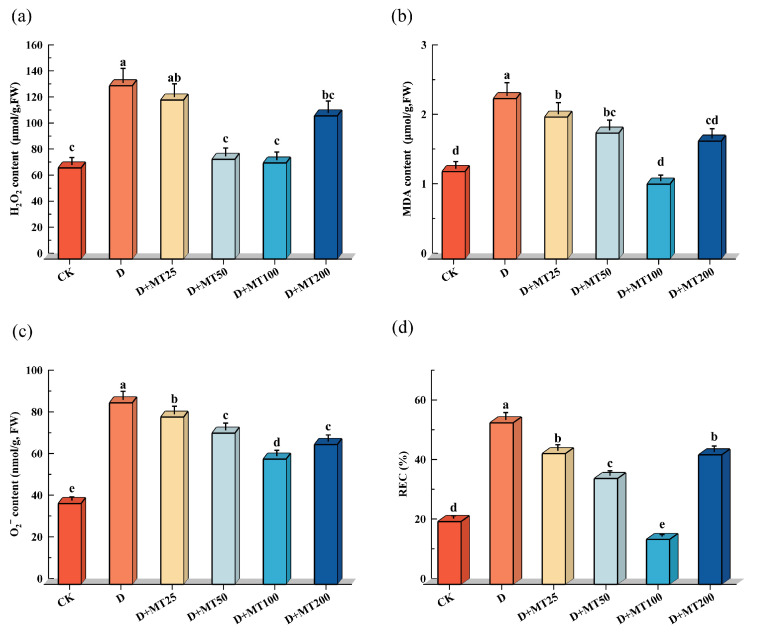
Effects of exogenous melatonin (MT) on the membrane lipid peroxidation in the leaves of *G. glabra* seedlings under drought stress. (**a**) Hydrogen peroxide (H_2_O_2_) content; (**b**) Malondialdehyde (MDA) content; (**c**) Superoxide anion (O_2_^−^) content; (**d**) Relative electrical conductivity (REC). Different lowercase letters indicate significant differences between different treatments, (Mean ± SD, *p* < 0.05).

Hydrogen peroxide (H_2_O_2_) and superoxide anion (O_2_^−^) are byproducts of cellular metabolism, and their accumulation serves as an indicator of a plant’s ROS-scavenging capacity under stress conditions. Melatonin, a potent antioxidant molecule, is known for its high efficiency in scavenging ROS. In this study, drought treatment significantly increased the levels of H_2_O_2_ and O_2_^−^ in *G. glabra* seedlings compared with CK, with respective increases of 90.25% and 124.56%. However, exogenous application of melatonin under drought condition markedly reduced ROS level. Specifically, treatment with 100 μM melatonin (D + MT100 treatment) decreased H_2_O_2_ and O_2_^−^ contents by 44.61% and 31.01%, respectively, compared with the D treatment (Figure 6a,c).

Compared with the CK, drought stress (D treatment) seriously increased the malondialdehyde (MDA) content and relative electrolyte conductivity (REC) in *G. glabra* leaves by 83.27% and 156.96%, respectively. However, the D + MT100 treatment effectively reduced the both parameters by 53.27% and 72.19%, respectively, compared with the D treatment. These results suggest that melatonin significantly mitigates drought-induced oxidative damage and helps maintain cell membrane stability (Figure 6b,d).

These results indicate that drought stress induces significant accumulation of reactive oxygen species (ROS), as well as increases in malondialdehyde (MDA) content and relative electrolyte conductivity (REC) in *G. glabra*, reflecting enhanced oxidative damage and membrane lipid peroxidation. However, exogenous application of melatonin effectively alleviated oxidative stress, with the 100 μM melatonin treatment showing the most pronounced mitigation effect.

### 2.6. Effects of Exogenous Melatonin on Osmoregulatory Substance Accumulation

**Figure 7:**
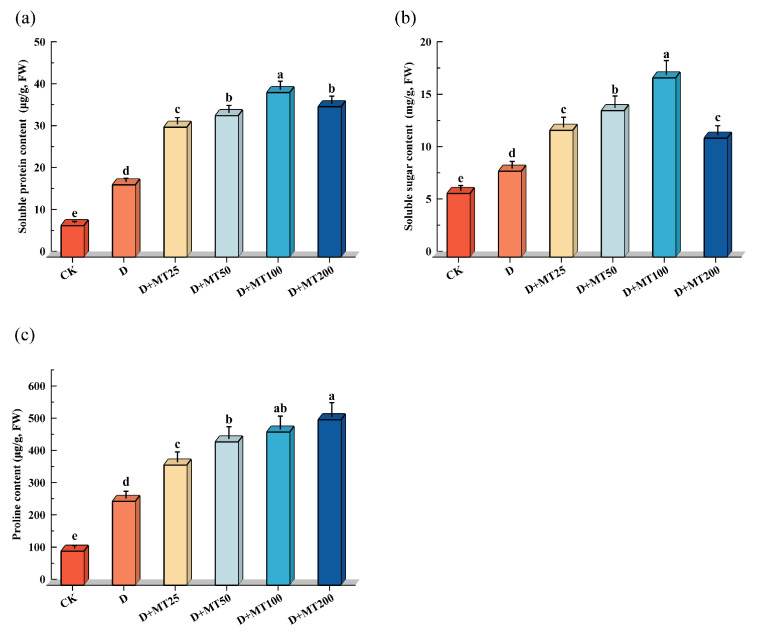
Effects of exogenous melatonin (MT) on the osmoregulation substance content in the leaves of *G. glabra* seedlings under drought stress. (**a**) Soluble protein content; (**b**) Soluble sugar content; (**c**) Proline content. Different lowercase letters indicate significant differences between different treatments, (Mean ± SD, *p* < 0.05).

Under drought treatment, the contents of soluble protein, soluble sugar, and proline in *G. glabra* seedlings increased by 129.57%, 35.02% and 146.85%, respectively, compared with CK, indicating that drought stress triggered the accumulation of osmoregulatory substances to maintain cellular osmotic balance (Figure 7). Exogenous application of melatonin under drought condition further enhanced the accumulation of these osmolytes. Among them, soluble protein and soluble sugar showed the most pronounced responses under 100 μM melatonin treatment, with increases of 127.35% and 108.72%, respectively. However, proline content exhibited a trend of continuous increase, and application of 200 μM melatonin resulted in a 97.25% increase compared with D treatment.

### 2.7. Effects of Exogenous Melatonin on the Accumulation of Secondary Metabolites

Under drought stress, the concentrations of glycyrrhizic acid, liquiritin, glabridin, and liquiritigenin in *G. glabra* roots decreased by 17.16%, 20.31%, 23.45% and 34.40%, respectively, compared with CK, while the concentration of isoliquiritigenin showed no significant change (Table 1). In addition, drought stress also led to reductions in the per-plant content of these 5 compounds by 63.17%, 64.63%, 65.96%, 70.00% and 60.10%, respectively (Table 2), indicating that water deficit negatively impacted both the concentration and total accumulation of the 5 secondary metabolites. Under drought condition, application of 100 μM melatonin significantly promoted the accumulation of these metabolites in *G. glabra* roots. Compared with D treatment, the concentrations of the 5 substances increased by 60.51%, 72.08%, 182.03%, 83.86% and 30.68%, respectively. Notably, these concentrations also showed significant higher than those of the CK, increasing by 32.98%, 37.11%, 115.96%, 20.61% and 30.68%, respectively. Furthermore, the per-plant contents of these five metabolites in the D + MT100 group were 3.8, 4.1, 6.7, 4.3. and 3.2 times as high as those in the D group, respectively.

### 2.8. Main Factors Affecting Yield and Quality of the Licorice Root

**Figure 8:**
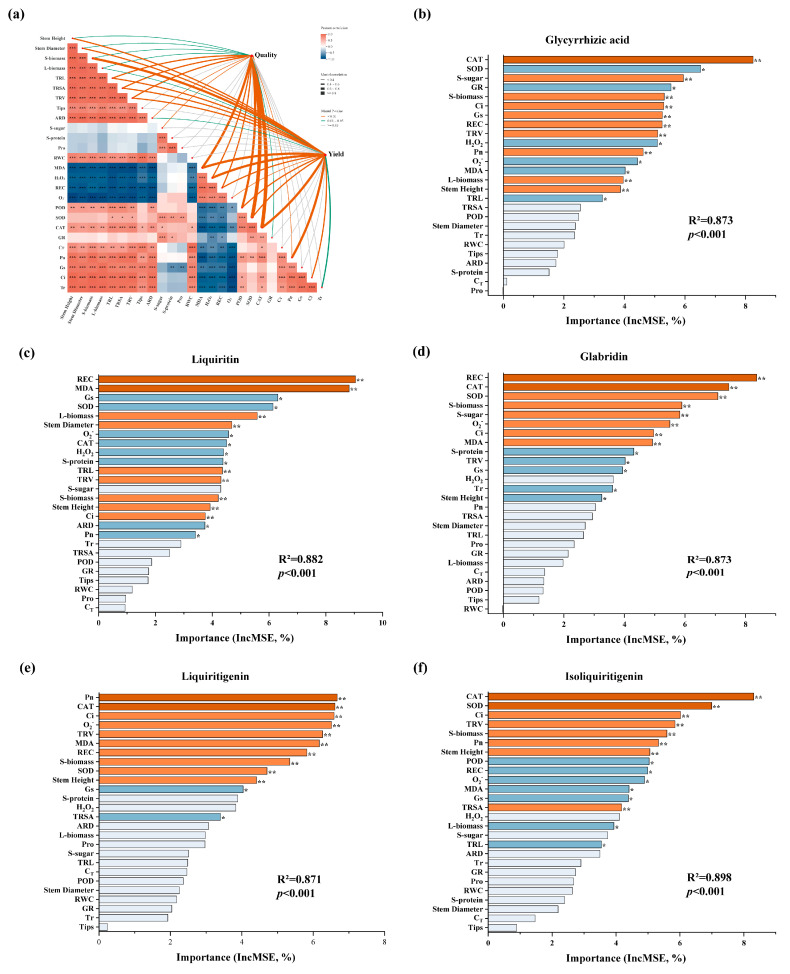
(**a**) Mantel test and correlation analysis of exogenous melatonin on the yield, quality, and physiological indexes of *G. glabra* under drought stress; Random forest models to predict the key physiological factors affecting the content of (**b**) glycyrrhizic acid, (**c**) liquiritin, (**d**) glabridin, (**e**) liquiritigenin and (**f**) isoliquiritigenin per plant. *, ** and *** indicate the significance level at *p* < 0.05, *p* < 0.01 and *p* < 0.001.

To explore the interactive mechanisms among growth, physiological responses, contents of secondary metabolites and root yield of *G. glabra*, mantel test was employed to analyze the structural correlations among the various indicators. In addition, random forest model was constructed to predict the key factors influencing the per-plant contents of the five compounds. Quality indicators included both the concentrations and per-plant contents of the five secondary metabolites: glycyrrhizic acid, liquiritin, glabridin, liquiritigenin, and isoliquiritigenin. Yield was represented by root biomass. The results of Mantel test showed that quality indicators were strongly correlated with the activities of antioxidant enzymes (POD, SOD, and CAT) and the level of membrane lipid peroxidation (MDA). Root yield was strongly associated with CAT activity, net photosynthetic rate (Pn), and stem biomass (Figure 8a). As shown in the Pearson correlation heatmap of the lower-left part of Figure 8a, growth indicators (Stem Height, Stem Diameter), root development parameters (TRL, TRSA, TRV, ARD, and Tips) and organ biomass (R/S/L-biomass) exhibited strong positive correlation with each other. Membrane lipid peroxidation parameters (MDA, H_2_O_2_, O_2_^−^, and REC) exhibited strong positive correlations among themselves and significant negative correlations with growth traits. In addition, the activities of antioxidant enzymes (POD, SOD, and CAT) were negatively correlated with ROS levels (H_2_O_2_ and O_2_^−^), highlighting the role of antioxidant enzymes in scavenging the reactive oxygen species (ROS) and stabilizing the cellular membrane system.

The key factors influencing the per-plant content of each active compound were revealed by the random forest model (Figure 8b–f). For accumulation of glycyrrhizic acid, CAT, SOD and GR activity and soluble sugar content were the main predictors. REC, MDA, Gs and SOD were the most influential factors for liquiritin content. The content of glabridin was primarily affected by REC, SOD, CAT, and stem biomass. Pn, CAT, Ci and O_2_^−^ played important roles for liquiritigenin synthesis, and the content of isoliquiritigenin was mainly influenced by activities of CAT and SOD and Ci and TRV.

## 3. Discussion

Water scarcity is becoming an increasingly severe environmental challenge worldwide, significantly affecting crops’ yield and quality of their economic organs [26]. In this study, we systematically examined the effects of drought stress on the growth, physiological and biochemical responses, yield, and quality formation of *G. glabra* seedlings, as well as the regulatory role of exogenous melatonin in mitigating drought-induced damage. Drought stress markedly inhibited licorice growth, as evidenced by significant reductions in stem height, stem diameter, and biomass accumulation in both aboveground and belowground organs (Figure 1), consistent with previous reports indicating that drought limits plant growth rate and carbon allocation [27,28]. As the primary organ responsible for water and nutrient uptake, root development was severely suppressed under drought condition [29], further reducing root yield and accumulation of key bioactive compounds. Unlike crops such as cotton (*Gossypium hirsutum* L.) and maize (*Zea mays* L.), whose economic organs are seeds and fruits, *G. glabra* relies on its roots, which are rich in active constituents, including glycyrrhizic acid, liquiritin, and glabridin [30]. Therefore, root growth and metabolite content are directly linked to economic returns. Our results revealed that drought stress significantly reduced root length, surface area and volume. However, melatonin application effectively counteracted these adverse effects, leading to substantial improvements in root architecture. This enhancement increased the root system’s capacity to explore and occupy soil space, thereby providing stronger structural support and more efficient water and nutrient acquisition for shoot development [23]. These results suggest that melatonin improves photosynthetic efficiency and carbon assimilation, promotes the allocation of photosynthates to roots, and ultimately enhances root growth and biomass accumulation [30].

Previous studies in *Arabidopsis thaliana* reported that melatonin suppresses primary root elongation while promoting lateral root initiation by inhibiting auxin biosynthesis and polar auxin transport [31]. Although melatonin and indole-3-acetic acid (IAA) both originate from tryptophan, accumulating evidence indicates that their regulatory roles in root development diverge at multiple levels [32]. Despite sharing a common precursor, the downstream actions of these two molecules are not identical. These findings suggest that melatonin is more likely to modulate IAA distribution and signaling rather than simply mimic [31,33]. In contrast, IAA regulates root development through the canonical TIR1/AFB-Aux/IAA-ARF signaling module, which establishes a distinct balance between primary and lateral root growth [34]. Notably, the interaction between melatonin and auxin exhibits strong species specificity. While melatonin inhibits primary root elongation in *A. thaliana*, our results in *G. glabra* demonstrate that melatonin, especially at 100 μM, markedly promotes both primary and lateral root development under drought stress. Such contrasting responses may arise from interspecific differences in melatonin sensitivity, auxin transport capacity, or the composition of melatonin-responsive regulatory networks [35]. In *G. glabra*, melatonin may preferentially stimulate primary root elongation to improve deep soil water acquisition under drought, thereby enhancing overall root architecture and stress tolerance.

Drought stress is one of the major abiotic factors limiting photosynthetic efficiency in plants. In this study, it significantly reduced the net photosynthetic rate (Pn), transpiration rate (Tr), stomatal conductance (Gs) and intercellular CO_2_ concentration (Ci) in *G. glabra* leaves (Figure 3), indicating that under drought conditions, licorice minimizes water loss by closing stomata; however, this also restricts CO_2_ uptake and utilization, thereby impairing carbon assimilation [36]. The simultaneous decline in Pn, Gs, and Ci suggests that limited CO_2_ availability is a key factor contributing to reduced photosynthetic performance. The decrease in Gs directly limits CO_2_ entry into the leaves, while the reduction in Ci further reflects the inhibitory effects of stomatal closure on carbon metabolism [37]. Notably, exogenous melatonin significantly improved gas exchange parameters under drought stress. Compared with the drought treatment (D treatment), melatonin application (D + MT treatment) markedly increased Gs, Tr, Ci, and Pn. Consistent with previous findings [38], the application of 100 μM exogenous melatonin enhanced total chlorophyll content and improve photosynthetic parameters, ultimately increasing photosynthetic efficiency. These results suggest that melatonin may alleviate CO_2_ limitation caused by water stress by regulating stomatal aperture, thereby enhancing photosynthetic performance [39]. This regulatory effect may involve melatonin-mediated modulation with the abscisic acid (ABA) signaling pathway [40] or modulation of aquaporin expression [41], which helps to maintain stomatal function and leaf water balance, and ultimately mitigating the negative impact of stomatal closure on photosynthesis.

Chlorophyll is an essential pigment in plant photosynthesis, playing a pivotal role in light absorption, energy transfer, and electron transport [42]. Drought stress accelerates the degradation of photosynthetic pigments by promoting the accumulation oxygen species (ROS), inhibiting chlorophyll biosynthetic enzyme activities, and disrupting chloroplast membrane integrity. These effects significantly impair photosynthetic efficiency, thereby reducing carbon assimilation and ultimately hindering plant growth and development [43]. In our study, exogenous melatonin application effectively counteracted the drought-induced decline in chlorophyll content, significantly enhancing chlorophyll levels in *G. glabra* leaves under drought conditions (Figure 3d). This finding is consistent with results in maize reported by Huang et al. [44], who demonstrated that melatonin alleviates drought-induced damage to the photosynthetic apparatus by modulating the antioxidant system and preserving chloroplast structure. Similarly, An et al. [45] reported that melatonin maintains chlorophyll homeostasis and delays leaf senescence by enhancing antioxidant enzyme activities (e.g., SOD, CAT, POD) and regulating the expression of chlorophyll metabolism-related enzymes. In addition, relative water content (RWC), a key indicator of plant drought tolerance, was significantly reduced in *G. glabra* under drought stress, indicating that water deficiency severely impairs cellular function. Melatonin treatment markedly restored RWC levels, suggesting a role in facilitating water uptake and retention under stress conditions [46].

The results of our study showed that under drought stress, the contents of hydrogen peroxide (H_2_O_2_) and superoxide anion (O_2_^−^) in *G. glabra* leaves increased by 94.70% and 122.89%, respectively, accompanied by an 83.56% rise in MDA content and a 181.38% increase in REC (Figure 6). These alterations indicate pronounced oxidative damage to cell membranes and a substantial enhancement in membrane permeability. This trend is consistent with findings in maize [47], where drought treatment markedly elevated H_2_O_2_ and MDA levels, ultimately reducing physiological activity and impaired photosynthetic capacity. Plants regulate ROS levels through an antioxidant enzyme system, comprising superoxide dismutase (SOD), peroxidase (POD), catalase (CAT), and glutathione reductase (GR) [48]. In this study, drought treatment markedly suppressed the activities of these enzymes in *G. glabra* (Figure 5). However, exogenous melatonin application notably enhanced the activities of these four enzymes. SOD catalyzes the dismutation of O_2_^−^ into H_2_O_2_ and O_2_, whereas CAT and POD further decompose H_2_O_2_ into H_2_O, thereby alleviating oxidative damage and sustaining cellular redox homeostasis [49]. GR, a key enzyme in the glutathione–ascorbate cycle, helps maintain the GSH/GSSG ratio and strengthening cellular antioxidant capacity [50]. Overall, our results indicate that drought stress compromises the antioxidant defense system in *G. glabra*, whereas melatonin application restores the activities of key antioxidant enzymes (SOD, CAT, POD, and GR), effectively suppresses membrane lipid peroxidation, reduces oxidative damage, and enhances membrane stability.

Under drought stress, plants synthesize various osmotic regulatory substances, including proline, soluble protein and soluble sugar. The accumulation of these compounds helps maintain cellular osmotic balance and supports water uptake, thereby enabling plants to cope with water-deficient environments [51]. In our study, the contents of soluble protein, soluble sugar, and proline in *G. glabra* seedlings were significantly elevated under drought stress, reflecting a positive physiological response to mitigate water deficit-induced damage (Figure 7). Exogenous melatonin application further strengthened this response. This finding is in strong agreement with a recent study in wheat [52], which demonstrated that melatonin promoted osmotic adjustment under drought. Similarly, Wang et al. [53] reported that melatonin significantly increased proline accumulation in maize by upregulating key genes involved in proline biosynthesis, such as Δ^1^-pyrroline-5-carboxylate synthase (P5CS), thereby enhancing drought tolerance and osmotic regulation.

Medicinal plants synthesize a wide array of secondary metabolites in response to complex and fluctuating environmental conditions, and these compounds hold substantial application value in the cosmetics, pharmaceuticals, and food. Glabridin, a flavonoid compound with notable whitening, antioxidant, and anti-inflammatory activities, is primarily derived from the roots of *G. glabra*, yet its naturally low abundance restricts its broader utilization in the pharmaceutical and cosmetic industries [54]. Glycyrrhizic acid, one of the most representative triterpenoid saponins in *Glycyrrhiza* species, exhibits diverse pharmacological activities, including anti-inflammatory, antiviral, and hepatoprotective effects, and serves as a key indicator for evaluating the medicinal quality of licorice [55]. In addition, other flavonoids such as liquiritin [56], liquiritigenin [57], and isoliquiritigenin [58] possess antioxidant, neuroprotective, and immunomodulatory activities. In this study, drought stress caused a substantial reduction in the contents of glycyrrhizic acid, liquiritin, liquiritigenin, and glabridin in *G. glabra* roots (Table 1 and Table 2). This decline may be attributed to the plant’s adaptive strategy under adverse conditions, whereby limited resources are preferentially allocated to essential physiological processes such as growth maintenance and oxidative damage repair rather than to secondary metabolite biosynthesis [59]. Moreover, drought-induced oxidative stress may compromise the structural integrity of key enzymes involved in secondary metabolism, such as UDP-glycosyltransferases and chalcone isomerase, thereby disrupting the normal functioning of the biosynthetic pathways [60]. Exogenous melatonin application significantly enhanced the levels of the five secondary metabolites in *G. glabra* roots under drought conditions, with some treatment groups even exceeding the control. This finding suggests that melatonin not only mitigates drought-induced physiological damage but may also activate specific biosynthetic pathways for flavonoids and triterpenoid saponins, thereby promoting the synthesis and accumulation of key active constituents such as liquiritigenin and glycyrrhizic acid. Mantel test results revealed significant and strong correlations between quality indicators and the activities of antioxidant enzymes (POD, SOD, CAT), as well as the membrane lipid peroxidation marker MDA (Figure 8a). These results indicate that the redox status of plants not only plays a critical role in stress defense, but also participate in quality formation by regulating secondary metabolic pathways. As a typical marker of oxidative damage, MDA reflects both the intensity of stress and the extent of cellular injury through its concentration changes [61]. Its strong alignment with quality variation patterns further highlights the central role of the stress response system in driving quality differentiation, providing a novel physiological perspective for quality regulation in licorice. Meanwhile, yield indicators were strongly correlated with CAT activity, net photosynthetic rate (Pn), and stem biomass, indicating that root yield formation relies on the coordinated regulation of antioxidant capacity, photosynthetic efficiency, and nutrient accumulation. The random forest model further identified the key factors influencing the per-plant content of five active compounds. Antioxidant enzymes such as CAT and SOD exhibited high importance across multiple models, while Ci, Gs, and soluble sugar also contributed (Figure 8). Overall, these findings demonstrated a tightly integrated physiological network linking antioxidant systems, photosynthetic performance, and the formation of quality and yield traits, thereby providing theoretical support and potential regulatory targets for improving the quality and productivity of *G. glabra* under drought stress.

## 4. Materials and Methods

### 4.1. Experimental Materials

The experiment was conducted on an open field located within the campus of Shihezi University (44°18′35″ N, 86°03′27″ E) in 2023–2024, with temperate continental climate. The seeds of *G. glabra* used in this study were provided by the Institute of Liquorice at Shihezi University. Melatonin (C_13_H_16_N_2_O_2_) was purchased from Macklin Biochemical Co., Ltd. (Shanghai, China), with a relative molecular weight of 232.28 and a purity greater than 98%. Standard compounds of glycyrrhizic acid (Cat No.: SG8600), liquiritin (Cat No.: SL8210), glabridin (Cat No.: SG8480), liquiritigenin (Cat No.: SL8200), and isoliquiritigenin (Cat No.: SI8220) were purchased from Solarbio Science & Technology Co., Ltd. (Beijing, China), each with a purity greater than 98%.

### 4.2. Experimental Methods

Uniform, healthy, and fully developed seeds of *G. glabra* were selected for the experiment. The seeds were pretreated with 98% sulfuric acid for 30 min to break dormancy, followed by rinsing with water. Subsequently, the seeds were evenly sown into plastic pots (diameter: 20 cm; height: 25 cm), with 10 seeds per pot and a sowing depth of 1 cm. A sandy loam substrate was used for cultivation, consisting of a 3:7 mixture of sand and loam. The sand was collected from the Gurbantunggut Desert, while the loam was obtained from the experimental field of Shihezi University. Each pot was filled with 8 kg of the mixed soil, which had the following properties: total nitrogen, phosphorus, and potassium concentrations of 0.315 g·kg^−1^, 0.131 g·kg^−1^, and 5.47 g·kg^−1^, respectively; available nitrogen, phosphorus, and potassium contents of 52.59 mg·kg^−1^, 5.23 mg·kg^−1^, and 50.04 mg·kg^−1^, respectively; and an organic matter content of 6.64 mg·kg^−1^. Fertilizers were applied when the seedlings reached the 3–4 true leaf stage (BBCH 13–14), following the protocol described by Dong et al. (2024) [62]. The phenological stages were determined according to the BBCH scale (Meier, 2001) [63]. Soil moisture content was regulated using the gravimetric method. During the seedling growth period, soil moisture was maintained at 80% of field capacity (FC). To minimize the influence of light and other environmental factors on plant growth, the positions of the pots were randomly rearranged once per week.

When the 6th true leaf emerged (BBCH 16), the drought stress treatment was initiated. Plants in the control group (CK) were maintained at 80% field capacity (FC) throughout the entire experiment, whereas those in the drought stress group (D) were maintained at 40% FC from the onset of treatment until the final harvest. To compensate for water loss due to transpiration, water was added daily at 22:00. After 7 days of drought treatment, melatonin irrigation was initiated while plants continued to be maintained at their respective FC levels (80% for CK and 40% for other groups). Specifically, 50 mL of melatonin solution at concentrations of 25 μM, 50 μM, 100 μM, or 200 μM was applied every 7 days for a total of six applications. Due to the high photosensitivity of melatonin [64], all applications were performed at night.

Each treatment was performed with three replicates, and the detail information of the treatments is presented in Table 3. Sampling was conducted on the 7th day after the final melatonin application. Physiological and biochemical parameters were subsequently measured for each treatment group. Upon completion of all measurements, the remaining plants were harvested.

### 4.3. Measurement of Plant Growth Parameters

#### 4.3.1. Stem Height and Diameter

Stem Height: For each group, ten plants were randomly selected, their stem height was measured, and the average value was calculated.

Stem Diameter: For each group, ten plants were randomly selected, their stem diameter at the junction between the plant and the soil surface was measured, and the average value was calculated.

#### 4.3.2. Morphological Parameters of the Root System

Three plants from each treatment group were randomly selected, and their roots were gently washed with running water to remove adhering sand particles. The root systems were then scanned using a digital scanner (Expression 11000XL; Epson, Suwa City, Japan). The scanned images were analyzed using the WinRHIZO root analysis system (WinRHIZO Pro2012b; Regent Instruments Inc., Quebec City, QC, Canada) to obtain key morphological parameters, including total root length (TRL), total root surface area (TRSA), total root volume (TRV), average root diameter (ARD), and total number of root tips (Tips).

#### 4.3.3. Biomass

After harvesting, each plant was separated into roots, stems, and leaves, which were individually placed into paper bags and oven-dried at 75 °C until a constant weight was achieved. The dry weights of roots, stems, and leaves were measured using a balance with 0.001 g precision (BS423S; Sartorius AG, Göttingen, Germany). The average values were calculated and recorded as root biomass (R-biomass), stem biomass (S-biomass), and leaf biomass (L-biomass), respectively.

### 4.4. Leaf Relative Water Content

Leaf relative water content (RWC) was determined using the oven-drying method [65]. Fresh leaves (0.1 g) were weighed to obtain the fresh weight (Wf). The samples were then fully immersed in distilled water until their weight no longer increased, the turgid weight (Wt) was recorded. Subsequently, the leaves were oven-dried at 75 °C until a constant weight was reached to determine the dry weight (Wd). RWC was calculated using the following formula:

RWC = (Wf − Wd)/(Wt − Wd) × 100%(1)

### 4.5. Photosynthetic Gas Exchange Parameters and Leaf Chlorophyll Content

The photosynthetically active radiation (PAR) was set to 1200 μmol·m^−2^·s^−1^, and the CO_2_ concentration was maintained at 400 μmol·L^−1^. Relative humidity and temperature were adjusted to match ambient conditions (22.2 ± 5.0% and 25.7 ± 4.0 °C, respectively). Measurements were taken from the third fully expanded leaf from the apex. Each group was measured in triplicate, and the average values were calculated.

Chlorophyll was extracted using the ethanol immersion method [66]. Fresh leaves were cut into small pieces (1 mm × 1 mm) and placed into 15 mL centrifuge tubes containing 96% ethanol. The tubes were tightly sealed and wrapped in aluminum foil to create a dark environment. During the 24 h extraction period, the tubes were gently shaken every hour. After extraction, the leaf residues were removed, and the volume of the extract was adjusted to 10 mL with 96% ethanol. The absorbance of the extract was measured at 649 nm (A_649_) and 665 nm (A_665_) using a multifunctional microplate reader (Multiskan SkyHigh 1550; Thermo Fisher Scientific, Waltham, MA, USA). Total chlorophyll content (C_T_) was calculated using the following formula. Each sample was measured in triplicate, and the average value was used for analysis.

Ca = (13.95 A_665_ − 6.88 A_649_) × [V/(1000 × W)] (2)

Cb = (24.96 A_649_ − 7.32 A_665_) × [V/(1000 × W)] (3)

C_T_ = Ca + Cb (4)

### 4.6. Antioxidant Enzyme Activities

The activities of superoxide dismutase (SOD), peroxidase (POD), catalase (CAT), and glutathione reductase (GR) in leaves were determined using commercial assay kits (SOD-BC0175, POD-BC0090, CAT-BC0200, GR-BC1165; Solarbio Science & Technology Co., Ltd., Beijing, China) according to the manufacturer’s protocols. Each group was measured in triplicate, and the average values were calculated for analysis.

### 4.7. Membrane Permeability and Lipid Peroxidation Indicators

Relative electrical conductivity (REC) of leaves was determined using the conductivity method. 1.0 g of fresh leaf samples were accurately weighed and placed into 15 mL centrifuge tubes containing 10 mL of deionized water. The samples were soaked at room temperature for 12 h. The electrical conductivity of the solution was then measured using a conductivity meter (Bante 540; Bante Instruments, Shanghai, China) and recorded as E1. Subsequently, the tubes were heated in a water bath at 100 °C for 30 min, cooled to room temperature, and the final conductivity was measured and recorded as E2. REC was calculated using the following formula:

REC = E1/E2 × 100%(5)

The contents of malondialdehyde (MDA), hydrogen peroxide (H_2_O_2_), and superoxide anion (O_2_^−^) in leaves were determined using commercial assay kits (MDA-BC0025, H_2_O_2_-BC3595, O_2_^−^-BC1295; Solarbio Science & Technology Co., Ltd., Beijing, China) according to the manufacturer’s instructions.

Each group was measured in triplicate, and the average values were calculated.

### 4.8. Osmoregulatory Substance Contents

Soluble sugar (S-sugar) content in leaves was determined using a commercial assay kit (BC0035; Solarbio Science & Technology Co., Ltd., Beijing, China) according to the manufacturer’s instructions.

Soluble protein (S-protein) content was measured using a plant-specific ELISA kit (JM-110029P1; Jingmei Biological Technology Co., Ltd., Yancheng, China).

Proline (Pro) content was determined using the acid-ninhydrin method. Absorbance of the reaction solution was measured at 520 nm using a multifunctional microplate reader.

Each group was determined in triplicate, and the average values were calculated for further analysis.

### 4.9. Secondary Metabolite Contents

Each of glycyrrhizic acid, liquiritin, glabridin, liquiritigenin, and isoliquiritigenin reference standards of 2.0 mg was put into 10 mL volumetric flasks, the mixed standard stock solution with a mass concentration of 200 µg·mL^−1^ was obtained by constant volume with methanol. The mixed standard solutions with concentrations of 1, 10, 100, 500, 1000, 5000, and 10,000 ng·mL^−1^ were obtained by gradient dilution with methanol. A calibration curve was constructed by plotting the peak area of quantitative ions (Y-axis) against the corresponding concentrations (X-axis, ng·mL^−1^), and the regression equation was established. The mass spectrometry conditions, regression equations, and correlation coefficients are listed in Table 4.

Dried root of *G. glabra* was ground into fine powder and passed through an 80-mesh sieve. A total of 0.5 g of root powder from each treatment group was accurately weighed and placed into a 10 mL centrifuge tube, followed by the addition of 5 mL of chromatographic-grade methanol. The samples were extracted at room temperature for 2 h using an ultrasonic extractor (KQ-300E; Kunshan Ultrasonic Instruments Co., Ltd., Kunshan, China). The extracts were centrifuged at 12,000 rpm for 15 min, and the supernatants were filtered through a 0.25 µm microporous membrane. The filtrates were analyzed using an Acquity UPLC system equipped with a Waters XEVO TQS triple quadrupole mass spectrometer to obtain chromatographic peak areas of glycyrrhizic acid, liquiritin, glabridin, liquiritigenin and isoliquiritigenin [67]. The concentrations of these five substances were calculated based on the aforementioned regression equation (Table 4). Each sample was determined in triplicate, and the average values were used for further analysis.

The average content of glycyrrhizic acid, liquiritin, liquiritigenin, isoliquiritigenin, and glabridin per plant (mg/plant) was calculated by multiplying the concentration of each compound by the average root biomass per plant.

### 4.10. Data Analysis

Statistical analyses were performed using SPSS 26.0 software (IBM Corp., New York, NY, USA). One-way analysis of variance (ANOVA) followed by least significant difference (LSD) tests was used to evaluate differences among treatments. Statistical significance was set at *p* < 0.05. All data are presented as mean ± standard deviation (SD). Graphs were generated using OriginPro 2025b software (Electronic Arts Inc., New York, NY, USA). The Mantel test and random forest model were implemented using R-4.5.1.

## 5. Conclusions

In summary, exogenous application of melatonin is an effective measure to enhance drought tolerance in *G. glabra* seedlings and improve the yield and quality of its economic organs. Among the tested concentrations, 100 μM melatonin exhibited the most pronounced promotive effects on growth, biomass accumulation, and secondary metabolite content under drought stress condition. As a novel plant growth regulator, melatonin shows promising potential for application under adverse environmental conditions. Future studies should integrate multi-omics approaches, including transcriptomics, metabolomics and proteomics, to systematically elucidate the regulatory mechanisms of melatonin across diverse species and stress scenarios. Such investigations will not only validate the generality of the present findings but also provide theoretical support for stress-resilient cultivation of licorice and facilitate its broader application in other economically important crops, thereby contributing to sustainable agricultural development.

## Figures and Tables

**Table 1:** The effects of exogenous melatonin on the concentration of five active components in the root system of *G. glabra* seedlings under drought stress.

Treatments	Concentrations of Active Components (μg/g)				
	Glycyrrhizic Acid	Liquiritin	Glabridin	Liquiritigenin	Isoliquiritigenin
CK	2021.21 ± 181.61 cd	998.78 ± 89.75 b	128.21 ± 11.52 c	128.78 ± 11.57 b	38.86 ± 3.49 bc
D	1674.28 ± 150.44 d	795.88 ± 71.51 c	98.14 ± 8.82 d	84.48 ± 7.59 d	35.99 ± 3.23 c
D + MT25	1838.04 ± 165.16 cd	869.04 ± 78.09 bc	120.47 ± 10.12 cd	100.98 ± 9.07 cd	39.78 ± 3.57 bc
D + MT50	2398.02 ± 215.47 ab	919.02 ± 82.58 bc	145.35 ± 13.06 bc	117.30 ± 10.54 bc	40.90 ± 3.68 abc
D + MT100	2687.79 ± 241.51 a	1369.52 ± 123.06 a	276.86 ± 24.88 a	155.31 ± 14.23 a	47.03 ± 4.23 a
D + MT200	2073.66 ± 186.33 bc	892.50 ± 80.20 bc	157.39 ± 14.14 b	158.28 ± 14.51 a	44.88 ± 4.03 ab

Different lowercase letters indicate significant differences between different treatments, (Mean ± SD, *p* < 0.05).

**Table 2:** The effects of exogenous melatonin on the 5 active ingredient content per plant in the root system of *G. glabra* seedlings under drought stress.

Treatments	Active Ingredient Content Per Plant (mg/Plant)				
	Glycyrrhizic Acid	Liquiritin	Glabridin	Liquiritigenin	Isoliquiritigenin
CK	7.79 ± 0.51 c	3.93 ± 0.33 b	0.50 ± 0.03 b	0.50 ± 0.04 c	0.15 ± 0.01 b
D	2.87 ± 0.19 e	1.39 ± 0.12 e	0.17 ± 0.01 d	0.15 ± 0.01 e	0.06 ± 0.01 d
D + MT25	5.22 ± 0.34 d	2.52 ± 0.21 d	0.35 ± 0.02 c	0.29 ± 0.03 d	0.11 ± 0.01 c
D + MT50	8.85 ± 0.57 b	3.46 ± 0.29 bc	0.54 ± 0.04 b	0.44 ± 0.04 c	0.15 ± 0.01 b
D + MT100	10.97 ± 0.71 a	5.70 ± 0.47 a	1.14 ± 0.08 a	0.64 ± 0.06 a	0.19 ± 0.02 a
D + MT200	7.00 ± 0.45 c	3.07 ± 0.26 c	0.54 ± 0.04 b	0.54 ± 0.05 b	0.15 ± 0.01 b

Different lowercase letters indicate significant differences between different treatments, (Mean ± SD, *p* < 0.05).

**Table 3:** Details of experimental treatments.

Code	Treatments Abbreviations	Soil Moisture Contents (% FC)	Melatonin (MT) Concentration (μM)
1	CK	80	0
2	D	40	0
3	D + MT25	40	25
4	D + MT50	40	50
5	D + MT100	40	100
6	D + MT200	40	200

Note: All plants were grown until 6 true leaf stage (BBCH 16), after which the treatments were applied.

**Table 4:** Optimized mass spectrometry conditions and regression equations for 5 compounds.

Compounds	Parent Ion (m·z^−1^)	Daughter Ion (m·z^−1^)	Ionization Mode	Retention Time (Min)	Regression Equation	Correlation Coefficients R^2^	Linear Over (ng·mL^−1^)
Glycyrrhizic acid	821.2	350.8 *	ESI−	4.83	Y = 409.2X − 3614.8	0.9998	10.5–5041.2
		112.9					
Liquiritin	416.9	254.9 *	ESI−	1.98	Y = 2560.9X + 1164.7	0.9994	0.9–987.2
		134.9					
Glabridin	322.9	134.9 *	ESI−	2.65	Y = 2428.9X + 845.1	0.9993	0.9–982.7
		200.9					
Liquiritigenin	257.1	136.9 *	ESI+	3.25	Y = 1237.1X + 2554.6	0.9917	0.8–1027.2
		146.9					
Isoliquiritigenin	256.9	136.9 *	ESI+	1.98	Y = 13,181.1X+ 49,642.4	0.9990	0.8–978.5
		146.9					

Note: * Stands for quantifier ions.

## Data Availability

Data are contained within the article.

## References

[1] Fu B., Stafford-Smith M., Wang Y., Wu B., Yu X., Lv N., Ojima D.S., Lv Y., Fu C., Liu Y. (2021). The Global-DEP conceptual framework—Research on dryland ecosystems to promote sustainability. Curr. Opin. Environ. Sustain..

[2] Tariq A., Sardans J., Zeng F., Graciano C., Hughes A.C., Farré-Armengol G., Peñuelas J. (2024). Impact of aridity rise and arid lands expansion on carbon-storing capacity, biodiversity loss, and ecosystem services. Glob. Change Biol..

[3] Gupta A., Rico-Medina A., Caño-Delgado A.I. (2020). The physiology of plant responses to drought. Science.

[4] Ali S., Mir R.A., Haque M.A., Danishuddin, Almalki M.A., Alfredan M., Khalifa A., Mahmoudi H., Shahid M., Tyagi A. (2025). Exploring physiological and molecular dynamics of drought stress responses in plants: Challenges and future directions. Front. Plant Sci..

[5] Qiao M., Hong C., Jiao Y., Hou S., Gao H. (2024). Impacts of drought on photosynthesis in major food crops and the related mechanisms of plant responses to drought. Plants.

[6] Nayak J.K., Mohanty D., Mahapatra D., Mondal S., Mohanty A., Sangeeta S., Nayak G., Senapaty J., Panda B., Nayak J.K. (2025). Flavonoids: A natural shield of plants under drought stress. Plant Secondary Metabolites Occurrence, Structure and Role.

[7] Yang X., Lu M., Wang Y., Wang Y., Liu Z., Chen S. (2021). Response mechanism of plants to drought stress. Horticulturae.

[8] Babich O., Ivanova S., Ulrikh E., Popov A., Larina V., Frolov A., Prosekov A. (2022). Study of the chemical composition and biologically active properties of *Glycyrrhiza glabra* extracts. Life.

[9] Lim T.K. (2015). *Glycyrrhiza* *glabra*. Edible Medicinal and Non-Medicinal Plants.

[10] Wahab S., Annadurai S., Abullais S.S., Das G., Ahmad W., Ahmad M.F., Kandasamy G., Vasudevan R., Ali M.S., Amir M. (2021). *Glycyrrhiza glabra* (licorice): A comprehensive review on its phytochemistry, biological activities, clinical evidence and toxicology. Plants.

[11] Nazari S., Rameshrad M., Hosseinzadeh H. (2017). Toxicological effects of *Glycyrrhiza glabra* (licorice): A review. Phytother. Res..

[12] Guo L., Katiyo W., Lu L., Zhang X., Wang M., Yan J., Ma X., Yang R., Zou L., Zhao W. (2018). Glycyrrhetic acid 3-O-mono-β-d-glucuronide (GAMG): An innovative high-potency sweetener with improved biological activities. Compr. Rev. Food Sci. Food Saf..

[13] Goyal P., Manzoor M.M., Vishwakarma R.A., Sharma D., Dhar M.K., Gupta S. (2020). A comprehensive transcrip-tome-wide identification and screening of WRKY gene family engaged in abiotic stress in *Glycyrrhiza glabra*. Sci. Rep..

[14] Eghlima G., Tafreshi Y.M., Aghamir F., Ahadi H., Hatami M. (2025). Regional environmental impacts on growth traits and phytochemical profiles of *Glycyrrhiza glabra* L. for enhanced medicinal and industrial use. BMC Plant Biol..

[15] Godoy F., Olivos-Hernández K., Stange C., Handford M. (2021). Abiotic stress in crop species: Improving tolerance by applying plant metabolites. Plants.

[16] Han Y., Hou Z., Zhang X., Yan K., Liang Z., He Q. (2022). Important changes in germination, seedling tolerance, and active components content due to drought stress on three licorice (*Glycyrrhiza*) species. Ind. Crops Prod..

[17] Zhu L., Li A., Sun H., Li P., Liu X., Guo C., Zhang Y., Zhang K., Bai Z., Dong H. (2023). The effect of exogenous melatonin on root growth and lifespan and seed cotton yield under drought stress. Ind. Crops Prod..

[18] Guo Y., Huang G., Guo Q., Peng C., Liu Y., Zhang M., Li Z., Zhou Y., Duan L. (2023). Increase in root density induced by coronatine improves maize drought resistance in North China. Crop J..

[19] Debnath B., Islam W., Li M., Sun Y., Lu X., Mitra S., Hussain M., Liu S., Qiu D. (2019). Melatonin mediates enhancement of stress tolerance in plants. Int. J. Mol. Sci..

[20] Arnao M.B., Hernández-Ruiz J. (2019). Melatonin: A new plant hormone and/or a plant master regulator?. Trends Plant Sci..

[21] Wang P., Sun X., Li C., Wei Z., Liang D., Ma F. (2013). Long-term exogenous application of melatonin delays drought induced leaf senescence in apple. J. Pineal Res..

[22] Yang K., Sun H., Liu M., Zhu L., Zhang K., Zhang Y., Li A., Zhang H., Zhu J., Liu X. (2023). Morphological and physiological mechanisms of melatonin on delaying drought-induced leaf senescence in cotton. Int. J. Mol. Sci..

[23] Michałek M., Ogrodowicz P., Kempa M., Kuczyńska A., Mikołajczak K. (2025). Melatonin in crop plants: From biosynthesis through pleiotropic effects to enhanced stress resilience. J. Appl. Genet..

[24] Xu L., Yang W., Guo H., Liu C., Chen A., Ahmad S.A., Wang X. (2025). Transcriptome analysis of grape root to determine the regulatory network response to melatonin. Hortic. Plant J..

[25] Fozi V., Esmaeili H., Alizadeh A., Eghlima G., Mirjalili M.H. (2024). The interaction effect of water deficit stress and seaweed extract on phytochemical characteristics and antioxidant activity of licorice (*Glycyrrhiza glabra* L.). Front. Plant Sci..

[26] Shahroudi E., Zarinkamar F., Rezayian M. (2023). Putrescin modulates metabolic and physiological characteristics of *Thymus daenensis* under drought stress. Sci. Hortic..

[27] Aili R., Deng Y., Yang R., Zhang X., Huang Y., Li H., Jia S., Yu L., Zhang T. (2023). Molecular mechanisms of alfalfa (*Medicago sativa* L.) in response to combined drought and cold stresses. Agronomy.

[28] Ahmad Z., Waraich E.A., Akhtar S., Anjum S., Ahmad T., Mahboob W., Hafeez O.B.A., Tapera T., Labuschagne M., Rizwan M. (2018). Physiological responses of wheat to drought stress and its mitigation approaches. Acta Physiol. Plant..

[29] Ahmad H.M., Fiaz S., Hafeez S., Zahra S., Shah A.N., Gul B., Aziz O., Mahmood-Ur-Rahman, Fakhar A., Rafique M. (2022). Plant growth-promoting rhizobacteria eliminate the effect of drought stress in plants: A review. Front. Plant Sci..

[30] Kalra A., Goel S., Elias A.A. (2024). Understanding role of roots in plant response to drought: Way forward to climate-resilient crops. Plant Genome.

[31] Wang Q., An B., Wei Y., Reiter R.J., Shi H., Luo H., He C. (2016). Melatonin Regulates Root Meristem by Repressing Auxin Synthesis and Polar Auxin Transport in *Arabidopsis*. Front. Plant Sci..

[32] Yang L., You J., Li J., Wang Y., Chan Z. (2021). Melatonin Promotes *Arabidopsis* Primary Root Growth in an IAA-Dependent Manner. J. Exp. Bot..

[33] Ren S., Rutto L., Katuuramu D. (2019). Melatonin Acts Synergistically with Auxin to Promote Lateral Root Development through Fine Tuning Auxin Transport in *Arabidopsis thaliana*. PLoS ONE.

[34] Dharmasiri N., Dharmasiri S., Estelle M. (2005). The F-Box Protein TIR1 Is an Auxin Receptor. Nature.

[35] Zia S.F., Berkowitz O., Bedon F., Whelan J., Franks A.E., Plummer K.M. (2019). Direct Comparison of *Arabidopsis* Gene Expression Reveals Different Responses to Melatonin versus Auxin. BMC Plant Biol..

[36] Fu Y., Li P., Liang Y., Si Z., Ma S., Gao Y. (2024). Effects of exogenous melatonin on wheat quality under drought stress and rehydration. Plant Growth Regul..

[37] Cornic G. (2000). Drought stress inhibits photosynthesis by decreasing stomatal aperture–not by affecting ATP synthesis. Trends Plant Sci..

[38] Zhao Q., Zheng X., Wang C., Wang Q., Wei Q., Liu X., Liu Y., Chen A., Jiang J., Zhao X. (2025). Exogenous melatonin improves drought tolerance by regulating the antioxidant defense system and photosynthetic efficiency in fodder soybean seedlings. Plants.

[39] Zhao C., Guo H., Wang J., Wang Y., Zhang R. (2021). Melatonin enhances drought tolerance by regulating leaf stomatal behavior, carbon and nitrogen metabolism, and related gene expression in maize plants. Front. Plant Sci..

[40] Waseem M., Hasan M.M., Hazzazi Y., Alharbi B.M., Ghani M.U., Ahmad P., Carriquí M. (2025). Potential mechanisms for the rapid post-drought reversal of ABA-induced stomatal closure by melatonin, 5-aminolevulinic acid, and brassinosteroids. Photosynthetica.

[41] Ding L., Fox A.R., Chaumont F. (2024). Multifaceted role and regulation of aquaporins for efficient stomatal movements. Plant Cell Environ..

[42] Arnao M.B., Hernández-Ruiz J. (2009). Protective effect of melatonin against chlorophyll degradation during the senescence of barley leaves. J. Pineal Res..

[43] Lin S., Song X.F., Mao H.T., Li S.Q., Gan J.Y., Yuan M., Zhang Z.W., Yuan S., Zhang H.Y., Su Y.Q. (2022). Exogenous melatonin improved photosynthetic efficiency of photosystem II by reversible phosphorylation of thylakoid proteins in wheat under osmotic stress. Front. Plant Sci..

[44] Huang B., Chen Y.E., Zhao Y.Q., Ding C.B., Liao J.Q., Hu C., Zhou L.J., Zhang Z.W., Yuan S., Yuan M. (2019). Exogenous melatonin alleviates oxidative damages and protects photosystem II in maize seedlings under drought stress. Front. Plant Sci..

[45] An W., Wang G., Dou J., Zhang Y., Yang Q., He Y., Tang Z., Yu J. (2025). Protective mechanisms of exogenous melatonin on chlorophyll metabolism and photosynthesis in tomato seedlings under heat stress. Front. Plant Sci..

[46] Imran M., Latif Khan A., Shahzad R., Aaqil Khan M., Bilal S., Khan A., Kang S.M., Lee I.J. (2021). Exogenous melatonin induces drought stress tolerance by promoting plant growth and antioxidant defence system of soybean plants. AoB Plants.

[47] Ahmad S., Kamran M., Ding R., Meng X., Wang H., Ahmad I., Fahad S., Han Q. (2019). Exogenous melatonin confers drought stress by promoting plant growth, photosynthetic capacity and antioxidant defense system of maize seedlings. PeerJ.

[48] Rao M.J., Duan M., Ikram M., Zheng B. (2025). ROS regulation and antioxidant responses in plants under air pollution: Molecular signaling, metabolic adaptation, and biotechnological solutions. Antioxidants.

[49] Rao M.J., Duan M., Zhou C., Jiao J., Cheng P., Yang L., Wei W., Shen Q., Ji P., Yang Y. (2025). Antioxidant defense system in plants: Reactive oxygen species production, signaling, and scavenging during abiotic stress induced oxidative damage. Horticulturae.

[50] Noctor G., Cohen M., Trémulot L., Châtel-Innocenti G., Van Breusegem F., Mhamdi A. (2024). Glutathione: A key modulator of plant defence and metabolism through multiple mechanisms. J. Exp. Bot..

[51] Gururani M.A., Venkatesh J., Tran L.S.P. (2015). Regulation of photosynthesis during abiotic stress-induced photoinhibition. Mol. Plant.

[52] Li X., Liu J., Zhang C., Liu Z., Guo X., Li S., Li H., Liu K., Li K., Ding M. (2025). Melatonin promotes yield increase in wheat by regulating its antioxidant system and growth under drought stress. Biology.

[53] Wang Y., Zhou W., Wang Z., Gao S., Zhang R. (2025). Integrated metabolome, transcriptome and physiological analyses of melatonin-induced drought responses in maize roots and leaves. Plant Growth Regul..

[54] Zhang J., Wu X., Zhong B., Liao Q., Wang X., Xie Y., He X. (2023). Review on the diverse biological effects of glabridin. Drug Des. Devel. Ther..

[55] Nascimento M.H.M.d., de Araújo D.R. (2022). Exploring the pharmacological potential of glycyrrhizic acid: From therapeutic applications to trends in nanomedicine. Future Pharmacol..

[56] Liao L., Zhang Z. (2022). Liquiritin relieves oxygen-glucose reperfusion-induced neuronal injury via inhibition of the p38MAPK/NF-κB signaling pathway. Rev. Bras. Farmacogn..

[57] Ramalingam M., Kim H., Lee Y., Lee Y.I. (2018). Phytochemical and pharmacological role of liquiritigenin and isoliquiritigenin from Radix Glycyrrhizae in human health and disease models. Front. Aging Neurosci..

[58] Nihal P.M., Mohapatra D., Manir A.M.A.A., Mehra A., Sutrapu S., Harish V., Mohd S. (2025). Unveiling the essence of isoliquiritigenin: Exploring its chemistry, pharmacokinetics, and pharmacological potential. Chem. Afr..

[59] Krasensky J., Jonak C. (2012). Drought, salt, and temperature stress-induced metabolic rearrangements and regulatory networks. J. Exp. Bot..

[60] Wang F., Ren G., Li F., Qi S., Xu Y., Wang B., Yang Y., Ye Y., Zhou Q., Chen X. (2018). A chalcone synthase gene AeCHS from Abelmoschus esculentus regulates flavonoid accumulation and abiotic stress tolerance in transgenic Arabidopsis. Acta Physiol. Plant..

[61] Zuo G., Mei W., Feng N., Zheng D. (2025). Photosynthetic performance index (PIabs) and malondialdehyde (MDA) content determine rice biomass under combined salt stress and prohexadione-calcium treatment. BMC Plant Biol..

[62] Dong X., Ma X., Zhao Z., Ma M. (2024). Exogenous betaine enhances salt tolerance of *Glycyrrhiza uralensis* through multiple pathways. BMC Plant Biol..

[63] Meier U. (2001). Growth Stages of Mono- and Dicotyledonous Plants.

[64] Boccalandro H.E., González C.V., Wunderlin D.A., Silva M.F. (2011). Melatonin levels, determined by LC-ESI-MS/MS, fluctuate during the day/night cycle in *Vitis vinifera* cv. Malbec: Evidence of its antioxidant role in fruits. J. Pineal Res..

[65] Chang Y., Zhang J., Bao G., Yan B., Qu Y., Zhang M., Tang W. (2021). Physiological responses of highland barley seedlings to NaCl, drought, and freeze-thaw stress. J. Plant Growth Regul..

[66] Zhong Y., Liu C., Wei B., Zhang J., An Y., Wang L. (2023). Exogenous 5-Aminolevulinic acid promotes osmotic stress tolerance of walnuts by modulating photosynthesis, osmotic adjustment and antioxidant systems. Forests.

[67] Shang M., Jia T., Ma M. (2025). Alleviation of salt stress in *Glycyrrhiza uralensis* by lanthanum nitrate: A predictive modeling approach. Front. Plant Sci..

